# The serotonergic system dysfunction in diabetes mellitus

**DOI:** 10.3389/fncel.2022.899069

**Published:** 2022-07-14

**Authors:** Yan Cai, Xiaolong Li, Hongli Zhou, Jiyin Zhou

**Affiliations:** National Drug Clinical Trial Institution, Second Affiliated Hospital, Army Medical University, Chongqing, China

**Keywords:** serotonin, 5-HT, diabetes, neuropathy, 5-HT receptor, hyperglycemia

## Abstract

Most peripheral serotonin (5-HT) is synthesized in enterochromaffin cells, and most circulating 5-HT is stored in platelets. As a monoamine, 5-HT has several functions in various non-neuronal and neuronal systems. In the central nervous system, it functions as a neurotransmitter to modulate feeding behavior and mood. Numerous clinical trials have focused on increasing 5-HT activation in the central nervous system, including those involving anti-obesity drugs currently in the market, although severe side effects on peripheral system can lead to the withdrawal of certain drugs. Recent studies have revealed that both the peripheral and central serotonergic systems play a vital role in diabetes and its complications. This review summarizes the roles of the serotonergic system in blood glucose regulation, diabetic macroangiopathy, diabetic peripheral neuropathy, and diabetic encephalopathy, indicating its potential clinical significance as a therapeutic target for the treatment of diabetes and its complications.

## Introduction

5-hydroxytryptamine (5-HT), also known as serotonin, has numerous functions in non-neuronal and neuronal systems. 5-HT was discovered in 1918 and was considered to be a vasoconstrictor stored in platelets. In 1937, 5-HT was discovered in enterochromaffin cells of the gastrointestinal tract and was called enteramine, since it was considered to induce smooth muscle contraction in the intestine (Erspamer and Asero, 1952). 5-HT was reported to be a neurotransmitter in 1952 (Brodie and Shore, 1957), and has been linked to appetite, behavior, mood, and sleep cycle regulation (Zucker, 1944). It is now known to function as a mitogen and hormone as well.

Most 5-HT in the periphery is synthesized by enterochromaffin cells (Sumara et al., 2012). As the rate-limiting enzyme for 5-HT production, tryptophan hydroxylase causes the hydroxylation of tryptophan to synthesize 5-HT. The synthesis of 5-HT occurs in two steps. In the first step, tryptophan hydroxylase (the rate-limiting enzyme for 5-HT production) metabolizes tryptophan to 5-hydroxytryptophan. In the second step, 5-hydroxytryptophan is decarboxylated to 5-HT by a nonspecific enzyme (aromatic L-amino acid decarboxylase). Thus, 5-HT synthesis is modulated by the availability of tryptophan and the activity of tryptophan hydroxylase. One of the two tryptophan hydroxylase isoforms, tryptophan hydroxylase 1, is mainly expressed in the peripheral tissues and also in the pineal gland, while the other, tryptophan hydroxylase 2, is chiefly expressed in a subset of neurons of the enteric and central nervous system, including those in the raphe nuclei of the brain stem (Malek et al., 2005; Li et al., 2011; Yabut et al., 2019). Although tryptophan hydroxylase 1 is extensively expressed in peripheral tissues, more than 90% of the total 5-HT in the body is synthesized in the intestine (Berger et al., 2009). Most peripheral 5-HT is stored in platelets and controls the hemodynamics upon platelet activation. Several recent studies have shown that 5-HT can be produced in other peripheral tissues, including in the pancreas, heart, and adipose tissue, and plays a role in the manner of cell-autonomous (Kim et al., 2010; Ponicke et al., 2012; Oh et al., 2015). 5-HT also acts autonomously on various cell types in the gastrointestinal, hematopoietic, cardiovascular, and immune systems, and in the liver, bone, and placenta (Amireault et al., 2013). Within the enteric and central nervous systems, 5-HT is produced and stored in presynaptic neurons. As 5-HT cannot physically cross the blood-brain barrier, the peripheral serotonergic system is functionally separate from the central serotonergic system.

5-HT modulates many physiological and pathological processes *via* several membrane-bound 5-HT receptors; moreover, the biological functions of 5-HT are restricted by its uptake into cells *via* 5-HT transporters (Wade et al., 1996). More than 14 5-HT receptors belonging to seven families mediate the different functions of 5-HT (Mohammad-Zadeh et al., 2008). The 5-HT_3_ receptor is a ligand-gated cation channel, while all other 5-HT receptors are G-protein-coupled receptors (Peroutka and Howell, 1994; Reeves and Lummis, 2002). Recently, a novel detection technology was used in zebra fish model to show that 5-HT concentration significantly decreases in early diabetes, and that 5-HT can be used as an effective biomarker for the early diagnosis of diabetes (Khoshnevisan et al., 2021).

Several recent reviews have discussed the role of 5-HT and its receptors in neurological diseases and metabolism (Politis and Niccolini, 2015; Tu et al., 2015; Fakhoury, 2016; Oh et al., 2016; Lalut et al., 2017; Yohn et al., 2017; Yabut et al., 2019; Jones et al., 2020). Especially one review has summarized that 5-HT is produced in some peripheral tissues, including pancreas, adipose tissue, and liver; 5-HT and its receptors are involved in several metabolic pathways in peripheral tissues in the manner of cell-autonomous, such as insulin secretion and cell proliferation during development of β-cells, lipolysis in adipocytes, gluconeogenesis and glucose uptake in hepatocytes (Oh et al., 2016). So this review only summarizes the recent results regarding the roles of the peripheral and central serotonergic systems in diabetes and its complications, especially diabetic macroangiopathy, diabetic peripheral neuropathy, and diabetic encephalopathy.

## Roles of the peripheral serotonergic system in blood glucose level regulation

Obese humans synthesize and release more 5-HT from the proximal small intestine, which is strongly associated with glycemic control and higher body mass. The 5-HT produced in the intestine is believed to be a vital driver of pathogenesis in human obesity and dysglycemia (Young et al., 2018). Pharmacological or genetic reductions in gut 5-HT protect against diet-induced obesity, hepatic steatosis and glucose intolerance (Sumara et al., 2012; Crane et al., 2015; Oh et al., 2015; Choi et al., 2018), illustrating a causative role of elevated gut-derived 5-HT in driving metabolic dysfunctions. Both the density of enterochromaffin cells and the expression of tryptophan hydroxylase 1 are increased in human obesity (Young et al., 2018); however, mechanisms driving such changes remain unknown. Interactions between intestine-produced 5-HT and gut microbiota have recently been hypothesized to regulate host glucose metabolism. Pharmacological suppression or knockout of tryptophan hydroxylase to abolish intestinal 5-HT synthesis in mice, with or without antibiotic-related microbiota elimination, shows remarkable amelioration of glucose clearance (Martin et al., 2019). The amelioration in host glucose clearance caused by antibiotic-induced alterations in microbiota composition are dependent on the production of intestine-derived 5-HT (Martin et al., 2019).

**FIGURE 1:**
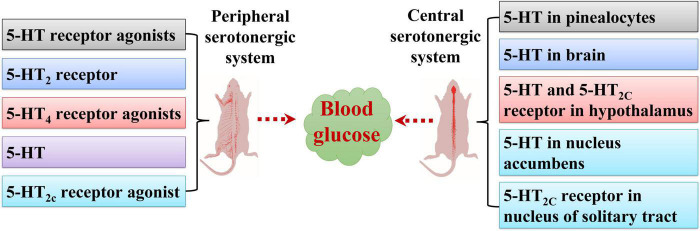
Effects of peripheral and central serotonergic systems on blood glucose levels. 5-HT, 5-HT_2_ receptor, 5-HT receptor agonists, 5-HT_4_ receptor agonists, and 5-HT_2c_ receptor agonists regulate blood glucose levels *via* the peripheral serotonergic system. 5-HT in the brain, pinealocytes, nucleus accumbens, and hypothalamus and 5-HT_2C_ receptors in the hypothalamus and nucleus of the solitary tract are involved in modulating blood glucose levels *via* the central serotonergic system.

With regard to insulin resistance, inhibition of intestine-secreted 5-HT improves glucose tolerance in high-fat diet-induced mice (Sumara et al., 2012). In hepatocytes, intestine-secreted 5-HT signaling *via* the 5-HT_2B_ receptor increases gluconeogenesis. Furthermore, intestine-derived 5-HT inhibits glucose uptake into hepatocytes through glucose transporter 2; hence, intestine-specific tryptophan hydroxylase 1 knockout mice and liver-specific 5-HT_2B_ receptor knockout mice show improved glucose tolerance compared to wild-type mice (Sumara et al., 2012). Glycemic regulation is also ameliorated in tryptophan hydroxylase 1 knockout mice, although the glucose uptake rate is the same as in the liver, muscle, and heart, suggesting that brown adipose tissue makes a major contribution to the increased basal metabolic rate (Crane et al., 2015). The effects of the peripheral and central serotonergic systems on blood glucose levels are shown in Table 1 and Figure 1.

**TABLE 1:** Summary of the role of serotonergic system in diabetes and its complication.

Serotonergic system	Target	Effects	References
Intestine-secreted 5-HT	5-HT_2B_ receptor in hepatocytes	Increases gluconeogenesis, increases serum glucose level	Sumara et al., 2012
		Inhibits glucose uptake into hepatocytes *via* glucose transporter 2 to increase serum glucose level	Sumara et al., 2012
8-hydroxy-2-(di-n-propylamino)tetralin 5-HT_1A_ receptor agonist	Central nervous system	Increased corticosterone leads to increased plasma glucose levels	Gehlert and Shaw, 2014
5-carboxamidotryptamine Peripherally acting nonselective 5-HT receptor agonist	Peripherally 5-HT_7_ receptor (>0.05 mg/kg)	Hyperglycemia in rats	Yamada et al., 1998
Mirtazapine 5-HT_2_ receptor antagonists	Peripheral	Worsens insulin sensitivity	Gilles et al., 2005
Fluoxetine Selective serotonin reuptake inhibitor	Peripheral	Ameliorates insulin sensitivity	Breum et al., 1995
Sertraline and paroxetine Selective serotonin reuptake inhibitor	Peripheral	Improves glycemic control	Goodnick et al., 1997; Ueno et al., 2010
Mosapride or prucalopride 5-HT_4_ receptor agonist	Pancreatic tissue and islet cells of rats *in vitro*	Increases insulin release; elevates the serum insulin level accompanied by a decrease in blood glucose	Chen et al., 2016
5-HT_4_ receptor	Nucleus accumbens	Reduces the physiological drive to eat	Jean et al., 2007
Lorcaserin 5-HT_2C_ receptor agonist	Peripheral	Loses weight and ameliorates glycemic parameters in prediabetes patients, and prevents progression to type 2 diabetes and accelerates reversion to normal blood glucose levels	Nesto et al., 2016
Lorcaserin 5-HT_2C_ receptor agonist	Brainstem nucleus of solitary tract	Decreasing food intake when activated	D’Agostino et al., 2018
Glucagon-like peptide 1 receptor	Dorsal raphe Hypothalamus	Body weight reduction, anorexia, and fat mass loss; 5-HT turnover and expression of 5-HT_2A_ and 5-HT_2C_ receptors	Anderberg et al., 2017
M-chlorophenylpiperazine 5-HT_2C_ receptor agonist	Peripheral	Ameliorates insulin sensitivity and glucose homeostasis	Berglund et al., 2013
Antagonists or genetic deletion of 5-HT_2C_ receptors	Pro-opiomelanocortin neurons	Compromises glucose homeostasis	Berglund et al., 2013
Transcutaneous auricular vagus nerve stimulation (2/15 Hz, 2 mA)		Inhibits the progression of nociceptive hypersensitivity in Zucker diabetic fatty rats, this advantageous impact on nociceptive behavior is associated with an increase in 5-HT plasma levels and upregulated expression of the 5-HT_1A_ receptor in the hypothalamus	Li et al., 2018
Duloxetine Inhibitor of serotonin and norepinephrine reuptake	Systemic or intrathecal injection	Alleviates tactile allodynia in diabetic rats	Mixcoatl-Zecuatl and Jolivalt, 2011

### 5-HT receptor agonists induce hyperglycemia in rats

5-carboxamidotryptamine, a peripherally acting nonselective 5-HT receptor agonist, induces significant hyperglycemia in rats at doses higher than 0.05 mg/kg. The above mentioned dose of 5-carboxamidotryptamine causing hyperglycemia can induce other pharmacological effects (drinking or hypophagia) in rats (Yamada et al., 1998). 5-carboxamidotryptamine-induced hyperglycemia is mediated by the facilitation of adrenaline secretion. Moreover, 5-carboxamidotryptamine causes hyperglycemia *via* 5-HT_7_ receptors, but not 5-HT_1A_, 5-HT_1B_, 5-HT_1D_, 5-HT_2_, 5-HT_3_, 5-HT_4_, and 5-HT_5_ receptors. This indicates that the 5-HT_7_ receptor may participate in adrenaline secretion like other 5-HT receptor subtypes (Yamada et al., 1998). 8-hydroxy-2-(di-*n*-propylamino)tetralin, a 5-HT_1A_ receptor agonist, causes hyperphagia and increases plasma glucose levels. It increases plasma corticosterone levels through a central nervous system mechanism, and increased corticosterone leads to higher plasma glucose levels (Gehlert and Shaw, 2014).

### Role of the 5-HT_2_ receptor in glucose metabolism

The 5-HT_2_ receptor participates directly in glucose metabolism in humans (Gilles et al., 2005). This is closely related to the effects of pharmacological agents that function as 5-HT_2_ receptor antagonists (such as mirtazapine), which can worsen insulin sensitivity and, in vulnerable patients or those with accompanying weight gain, may elevate the risk of impaired glucose tolerance and eventually diabetes. These observations confirm the hypothesis that serotonergic antidepressants directly deteriorate insulin sensitivity (Gilles et al., 2005). The 5-HT_2A_ receptor is expressed on both human skeletal muscle cells (Hajduch et al., 1999b) and in adult rat skeletal muscle (Hajduch et al., 1999a). It can stimulate rapid glucose uptake in rat skeletal muscle *in vitro*, particularly in L6 myotubes (Hajduch et al., 1999b). Therefore, several drugs may damage insulin sensitivity at the level of 5-HT_2_ receptor-mediated modulation of glucose transporters in muscle. To date, the antagonist property at 5-HT_2A_ receptors of clozapine and olanzapine have been associated with antipsychotic-induced diabetes (Henderson, 2002). This may be due to the obvious drug-related weight gain (Nasrallah, 2003), highlighting another disadvantageous effect of impaired insulin sensitivity on metabolism. However, some data suggest that all 5-HT_2_ receptor antagonists, including both these drugs, may pose a risk associated with insulin sensitivity (Gilles et al., 2005).

Fluoxetine, a selective serotonin reuptake inhibitor, induces the inhibition of 5-HT_2c_ receptors, and its short-term treatment is linked to weight loss; however, it also appears to ameliorate insulin sensitivity beyond the efficacy produced *via* weight loss by a likely stimulating effect on glycogen synthase activity in skeletal muscle (Breum et al., 1995). Sertraline and paroxetine, selective serotonin reuptake inhibitors, though lacking direct 5-HT_2c_ inhibiting properties, may also improve glycemic control (Goodnick et al., 1997; Paile-Hyvarinen et al., 2003).

### 5-HT_2C_ receptor agonists prevent type 2 diabetes

Lorcaserin (Belviq, lorcaserin hydrochloride), a 5-HT_2C_ receptor agonist, was the first drug approved by the U.S. Food and Drug Administration-to treat obesity in 2010 (2012). Several studies have analyzed the impact of lorcaserin on the progression from prediabetes to type 2 diabetes and on the restoration of prediabetes blood glucose levels to normal levels (Higgins et al., 2020). It can result in weight loss and ameliorate glycemic parameters in prediabetes patients, and can prevent progression to type 2 diabetes and accelerate reversion to normal blood glucose levels (Nesto et al., 2016). It has also been shown to ameliorate glycemic control in relation to weight loss in obese patients with type 2 diabetes (Burke et al., 2017). Brain pro-opiomelanocortin peptides are essential and adequate neurochemical mediators of lorcaserin’s glucoregulatory influence. Lorcaserin dose-dependently ameliorates glycemic control in type 2 diabetes mice without decreasing food intake or body weight. It needs functional melanocortin-4 receptors on cholinergic preganglionic neurons to trigger its effects on glucose homeostasis (Burke et al., 2017). In contrast, melanocortin-4 receptors on cholinergic preganglionic neurons do not affect lorcaserin’s influence on feeding, suggesting a diversity in the neurocircuitry underpinning lorcaserin’s curative glycemic and anorectic effects. A hyperinsulinemic-euglycemic clamp study revealed that lorcaserin decreases hepatic glucose synthesis, raises glucose disposal, and ameliorates insulin sensitivity (Burke et al., 2017). In its recently CAMELLIA-TIMI 61 trail (Scirica et al., 2019), there is a small raise in cancer in lorcaserin group, so the U.S. Food and Drug Administration asks the manufacturer to withdraw lorcaserin from the U.S. market in February 2021 (Mathai, 2021).

### 5-HT_4_ receptor agonists reduce blood glucose level

Gastrointestinal diseases, including constipation and gastroparesis, are common in diabetic patients. As an agonist of the 5-HT_4_ receptor, mosapride ameliorates these symptoms and controls the glycemic response (Ueno et al., 2010). The 5-HT_4_ receptor is expressed in β-cells in mice, rats, pigs, and humans. Stimulation of the 5-HT_4_ receptor with mosapride or prucalopride (a selective, high-affinity 5-HT_4_ receptor agonist) induces insulin release in both pancreatic tissue and islet cells of rats *in vitro*. In rats, it also elevates the serum insulin level accompanied by a decrease in blood glucose (Chen et al., 2016).

## Role of the central serotonergic system in eating and blood glucose regulation

Central serotonergic activity may modulate glucose metabolism *via* neuroendocrine effectors (Trento et al., 2010). There are obvious alterations in 5-HT levels in the prefrontal cortex, and in γ-aminobutyric acid and glutamate levels in the hippocampus in Spontaneously Diabetic Torii fatty rats compared with those in control rats (Sakimura et al., 2018). Hypothalamic 5-hydroxyindoleacetic acid concentrations in Goto-Kakizaki rats, a spontaneous diabetic model, were not different from those in control rats, whereas hypothalamic 5-HT concentrations in the streptozotocin-induced diabetic rats were significantly decreased (Gotoh et al., 2006). In addition, the expression of 5-HT_1A_ receptors in the hypothalamus can be modulated by serum glucose levels (Jhanwar-Uniyal et al., 1994).

### 5-HT in feeding and anorexigen action

Maintaining the energy balance requires modulation of the amount and timing of food intake, and eating disorders are emerging as a critical health issue in several developed countries. As confirmed in the central nervous system, 5-HT is a fundamental neurotransmitter regulating several physiological processes that affect food intake. The most important features of the serotonergic effects on energy balance pathways are linked to the regulation of the arcuate nucleus pro-opiomelanocortin and agouti-related peptide/neuropeptide Y neuronal populations. Previous studies have established that 5-HT hyperpolarizes and suppresses agouti-related peptide/neuropeptide Y neurons and decreases the suppressive drive onto pro-opiomelanocortin cells by stimulation of 5-HT_1B_ receptors. The 5-HT also activates pro-opiomelanocortin/cocaine- and amphetamine-regulated transcript neurons through activation of 5-HT_2C_ receptors (Heisler et al., 2006), which results in a reciprocal increase in α-melanocyte stimulating hormone secretion and decline in agouti-related peptide secretion at melanocortin 4 receptors in the target areas. Subsequent elevation of 5-HT neurotransmission also modulates the hypothalamic-pituitary-adrenal axis (HPA) upstream of the corticotropin-secreting hormone (Heisler et al., 2007). CP-809101 (a selective 5-HT_2C_ agonist) suppresses responding motivated by both food and nicotine in rats. CP-809101 inhibits the discriminative stimulus characteristics of nicotine with the same as Ro 60-0175 and lorcaserin (two structurally distinct 5-HT_2C_ receptor agonists). Behaviors including hypolocomotion, ptosis, and chewing become evident after treated with both higher doses of lorcaserin and CP-809101. These results indicate that the use of 5-HT_2C_ agonists as a therapeutic drug to treat nicotine dependence (Higgins et al., 2013).

The nucleus accumbens is a brain structure implicated in reward. Direct activation of 5-HT_4_ receptors in the nucleus accumbens suppresses the physiological initiation to feed and upregulates mRNA expression of cocaine- and amphetamine-regulated transcript in food-deprived and fed mice (Jean et al., 2007). Knockdown of 5-HT_4_ receptor with siRNA or injecting 5-HT_4_ receptor antagonist into the nucleus accumbens causes hyperphagia uniquely in fed but not in food-deprived mice. In both food-deprived and fed mice, this hyperphagia is not linked to alteration in mRNA expression of cocaine- and amphetamine-regulated transcript in the nucleus accumbens. 5-HT_4_ receptor regulates mRNA expression of cocaine- and amphetamine-regulated transcript into the nucleus accumbens through a cyclic adenosine monophosphate/protein kinase A signaling pathway (Jean et al., 2007). The nucleus accumbens-5-HT_4_ receptor/cocaine- and amphetamine-regulated transcript pathway tightly connects between hyperactivity and anorexia, indicating the presence of a fundamental functional unit susceptible to inhibit overeating linked to resting abiding by the rules of homeostasis (Jean et al., 2012).

### Decreased 5-HT levels in pinealocytes of diabetic pigs

Streptozotocin-induced diabetes significantly impacts sympathetic neurotransmission and the metabolism of melatonin synthesis-associated indoles in the pineal gland of pigs. The most prominent effect of diabetes on pig pinealocytes is the reduction in 5-HT levels. However, the synthesis of 5-HT is not influenced, since there is no change in 5-hydroxytryptophan content; therefore, two mechanisms that could result in the reduction in 5-HT content should be taken into account—increased utilization of 5-HT for *N*-acetylserotonin synthesis and insufficient storage of 5-HT (Lewczuk et al., 2018). The *N*-acetylserotonin level in the pineal gland of diabetic pigs is remarkably higher than that in control pigs; however, this is not reflected in an elevated melatonin content. Moreover, there were no discrepancies in the basal and adrenergic-induced secretion of melatonin and N-acetylserotonin between the pineal glands of control and diabetic pigs *in vitro* (Lewczuk et al., 2018).

### Elevating brain 5-HT levels to treat diabetes

Intranasal 5-HT administration reduces the body weight of diabetic rats and improves glucose tolerance, insulin-induced glucose utilization, and lipid metabolism (Derkach et al., 2015). Moreover, it restores the hormonal modulation of adenylyl cyclase activity in the hypothalamus and normalizes adenylyl cyclase activation by β-adrenergic agonists in the myocardium. The same administration induces metabolic and hormonal changes in control rats, some of which resemble those in type 2 diabetes, but to a lesser degree. The elevation of brain 5-HT content may be considered an effective method to treat type 2 diabetes and its complications (Derkach et al., 2015). Several other studies show that the intranasal delivery of peptides or proteins is a potential method to overcome the obstacles of the blood–brain barrier (Lochhead and Thorne, 2012). But the pathways and mechanisms of molecules delivery to central nervous system from the nasal passages still need completely understood.

### Role of hypothalamic 5-HT in hypoglycemic regulation

Insulin-induced hypoglycemia activates widespread 5-HT release in several forebrain areas, such as the ventromedial hypothalamus, perifornical hypothalamus, paraventricular hypothalamus, cerebral cortex, and paraventricular thalamic nucleus (Otlivanchik et al., 2015). In conscious rats, bilateral perifornical hypothalamic glucoprivation with 5-thioglucose significantly triggers adrenal medullary epinephrine secretion and feeding, while clamping perifornical hypothalamic glucose levels in the postprandial brain blunts the epinephrine response to hypoglycemia by 30%. The perifornical hypothalamus modulates adrenomedullary and feeding responses in the case of a metabolic emergency in freely behaving conscious rats. These responses are partly mediated by perifornical hypothalamic orexin neurons and 5-HT signaling (Otlivanchik et al., 2015). The perifornical hypothalamus has glucose-excited and glucose-inhibited neurons—glucose-excited neurons are principally excited, while glucose-inhibited neurons can be inhibited or excited by 5-HT at hypoglycemic glucose levels *in vitro*. 5-HT also stimulates lactate generation in hypothalamic astrocytes *in vitro*. Depleting perifornical hypothalamus 5-HT blunts the epinephrine (but not feeding) response to the focal perifornical hypothalamus and systemic glucoprivation, while raising the perifornical hypothalamus 5-HT level amplifies the epinephrine response to hypoglycemia by 32% (Otlivanchik et al., 2015). Perifornical hypothalamus 5-HT promotes the adrenomedullary response, and that perifornical hypothalamus orexin neurons change the electrophysiological properties of adrenal premotor neurons facilitating adrenaline release in response to glucopenia response to local and systemic glucose deficit (Korim et al., 2014). Selective serotonin reuptake inhibitors increase the counterregulatory response to acute hypoglycemia and avoid the blunting of the counterregulatory response after recurrent hypoglycemia in rats (Sanders et al., 2008) and humans (Briscoe et al., 2008a,b), indicating that 5-HT plays a vital role in mediating this response. These results suggest a critical role for 5-HT in strengthening the counterregulatory response to hypoglycemia. But it is still unknown how serotonergic system is activated in response to insulin-induced hypoglycemia. And whether serotonergic neurons are activated by either decreased glucose level or increased insulin level is still unknown.

### Role of nucleus accumbens 5-HT in blood glucose homeostasis

The nucleus accumbens is part of the reward circuitry that regulates feeding behavior. It contains glucose-sensing neurons (Papp et al., 2007) and provides input to the lateral hypothalamus (Groenewegen and Russchen, 1984), another region involved in glucose control (Yi et al., 2009; Morgan et al., 2015). Electrical stimulation of the shell nucleus accumbens using deep brain stimulation raises plasma levels of glucagon and glucose and stimulates neurons in the lateral hypothalamus (Diepenbroek et al., 2013), indicating a role for the shell nucleus accumbens in the modulation of glucose homeostasis. Locally infusing fluoxetine, a 5-HT reuptake inhibitor, in the shell nucleus accumbens of rats elevates blood glucose levels without overall altering glucoregulatory hormones, such as glucagon, insulin, and corticosterone. Its mechanism is not *via* the activity of the autonomic nervous system, since the glucagon level is not impacted. The effect on glucose levels might be due to the combined effects of higher endogenous glucose production and direct effect of 5-HT availability in the shell area of the nucleus accumbens on peripheral glucose uptake. Since peripheral glucose uptake is regulated by hypothalamus (Sudo et al., 1991) and the hypothalamus receives dense projections from the shell area of the nucleus accumbens (Sano and Yokoi, 2007). These results establish a role for the shell nucleus accumbens in systemic glucose metabolism and indicate that 5-HT may play a vital role in mediating these effects (Diepenbroek et al., 2017).

### 5-HT_2C_ receptors in the nucleus of the solitary tract and hypothalamus modulate food intake and glucose homeostasis

Selective stimulation of 5-HT_2C_ receptors in the nucleus of the solitary tract in the brainstem reduces feeding and is adequate to mediate acute food intake decrease induced by the use of lorcaserin, a 5-HT_2C_ receptor agonist, as an obesity medication (D’Agostino et al., 2018). A subpopulation of hypothalamic neurons in the brainstem nucleus of solitary tract resemble pro-opiomelanocortin neurons in the hypothalamic arcuate nucleus and co-express 5-HT_2C_ receptors and are stimulated by 5-HT_2C_ receptor agonists. Deletion of 5-HT_2C_ receptors in the hypothalamic arcuate nucleus inhibits the acute appetite-inhibitive function of lorcaserin; conversely, deletion in the hypothalamic arcuate nucleus prevents the total anorectic effect. These results indicate that 5-HT_2C_ receptors in the brainstem nucleus of the solitary tract represent a subpopulation of 5-HT_2C_ receptors that are capable of decreasing food intake when activated, and reveal that 5-HT_2C_ receptor agonists as obesity medications need pro-opiomelanocortin in the nucleus of the solitary tract and the arcuate nucleus to reduce food intake (D’Agostino et al., 2018).

Mice lacking 5-HT_2C_ receptors, especially in pro-opiomelanocortin neurons, show normal body weight but exhibit glucoregulatory defects, such as hyperglycemia, hyperglucagonemia, hyperinsulinemia, and insulin resistance (Berglund et al., 2013). In addition, these mice have no anorectic responses to serotonergic agents that inhibit appetite and gradually become hyperphagic and obese after being fed a high-fat/high-sugar diet. The demand for 5-HT_2C_ receptors in pro-opiomelanocortin neurons to maintain normal energy and glucose homeostasis is further indicated by the effects of 5-HT_2C_ knockout in pro-opiomelanocortin neurons in adult mice using a tamoxifen-inducible pro-opiomelanocortin-Cre system (Berglund et al., 2013). Treatment with m-chlorophenylpiperazine, a non-selective 5-HT_2C_ receptor agonist, ameliorates insulin sensitivity and glucose homeostasis, and antagonists [ritanserin (a 5-HT_2_ and 5-HT_1c_ antagonist) and MDL 72222 (a 5-HT antagonist)] or genetic deletion of 5-HT_2C_ receptors compromises glucose homeostasis (Berglund et al., 2013). These results show that 5-HT_2C_ receptor-expressing pro-opiomelanocortin neurons are required to regulate energy and glucose homeostasis and imply that pro-opiomelanocortin neurons are the target for the effect of 5-HT_2C_ receptor agonists on weight reduction and glycaemia regulation (Berglund et al., 2013). Postsynaptic receptors, such as 5-HT_1B_, 5-HT_2C_, and possibly 5-HT_2B_ receptors, are assumed to be directly stimulated by dexfenfluramine-induced 5-HT transporter-dependent and 5-HT_2B_ receptor-dependent 5-HT secretion (Banas et al., 2011).

5-HT_2C_ receptors expressed by pro-opiomelanocortin neurons of hypothalamic arcuate nucleus modulate food intake, energy homeostasis, and glucose metabolism. A subpopulation (about 25%) of the hypothalamic arcuate nucleus neurons, which areas are differ from leptin-activated, are depolarized by the 5-HT_2C_ receptors agonist (m-chlorophenylpiperazine) through activation of the putative transient receptor potential C channels (Sohn et al., 2011). With this consistent mechanism, to record the brainstem nucleus of the solitary tract neurons in a voltage clamp and treated with tetrodotoxin to antagonize the inhibitory effect of 5-HT, 5-HT, and lorcaserin excite the brainstem nucleus of the solitary tract neurons through the activation of a post-synaptic, mixed cationic current (D’Agostino et al., 2018).

### 5-HT as a substrate of glucagon-like peptide 1 to impact energy homeostasis

5-HT depletion impairs the effect of exendin-4, a glucagon-like peptide 1 analog, to reduce body weight in rats, indicating that 5-HT is a vital mediator of the energy balance-related effect of glucagon-like peptide 1 receptor stimulation. 5-HT turnover and expression of 5-HT_2A_ and 5-HT_2C_ receptors in the hypothalamus are also altered by glucagon-like peptide 1 receptor activation (Anderberg et al., 2017). The 5-HT_2A_ receptor is significantly associated with body weight reduction, anorexia, and fat mass loss caused by central glucagon-like peptide 1 receptor activation. Importantly, 5-HT_2A_ receptors in the brain are also needed for peripherally administered liraglutide, a glucagon-like peptide-1 receptor agonist, to decrease feeding and body weight (Anderberg et al., 2017). The dorsal raphe harbors the cell bodies of 5-HT-synthesizing neurons that provide 5-HT to the hypothalamic nuclei. Glucagon-like peptide 1 receptor activation in the dorsal raphe is sufficient to lower hypophagia and elevate the electrical activity of serotonergic neurons in the dorsal raphe. This identifies 5-HT as a novel pivotal neural substrate for glucagon-like peptide 1 in influencing energy homeostasis and amplifies the current map of brain regions affected by glucagon-like peptide 1 receptor activation (Anderberg et al., 2017).

## Role of the serotonergic system in diabetic macroangiopathy

5-HT is a significant vasoactive monoamine in the cardiovascular system. Reduction of 5-HT levels in platelets and elevation in plasma concentration may reflect increased secretion of 5-HT by hyperactive platelets. This elevation in plasma 5-HT levels may lead to the pathogenesis of vasospasm and atherosclerosis (Barradas et al., 1988), an important feature of cardiovascular diseases in diabetes (Forbes and Cooper, 2013).

Plasma 5-HT levels are elevated in diabetic patients, and this elevation is associated, at least in part, with platelet hyperfunction (Hasegawa et al., 2002). Although 5-HT-induced platelet aggregation is enhanced in diabetic patients, with an increase in serum advanced glycation end-product levels, there is no association between platelet aggregation and either fasting blood glucose or hemoglobin A_1c_ levels. 5-HT-stimulated platelet aggregation is dose-dependently increased by advanced glycation end-products. Adenosine diphosphate-activated platelet aggregation is also enhanced by advanced glycation end-products; moreover this increase is abolished by the action of sarpogrelate, a selective 5-HT_2*A*_ receptor antagonist (Hasegawa et al., 2002). These results suggest that advanced glycation end-products enhance platelet aggregation *via* the 5-HT receptor and may influence the development of thrombotic complications in diabetic patients (Hasegawa et al., 2002).

### 5-HT_2A_ receptors in diabetes-related vascular complications

5-HT, stimulated by damaged vascular endothelial cells, participates in the pathological process of vascular complications such as carotid artery contraction (Matsumoto et al., 2014) and arteriogenesis (Bir et al., 2008) in diabetes *via* the 5-HT_2A_ receptor. Sarpogrelate, a 5-HT_2A_ receptor antagonist, attenuates diabetic cardiovascular complications; it decreases the blood glucose concentration (Matsumoto et al., 2014), inhibits the production of vascular cell adhesion molecule-1 and intercellular adhesion molecule-1 (Su et al., 2013), and reduces 5-HT-triggered contraction in aortas *via* PI3K pathways (Matsumoto et al., 2014). Combined sarpogrelate and sustained-release basic fibroblast growth factor therapy is effective in inducing neovascularization to restore arteriogenesis and tissue blood perfusion in diabetic mice (Bir et al., 2008). High glucose levels increase intercellular adhesion molecule-1 expression and decrease superoxide dismutase activity; these effects are partially abolished by sarpogrelate hydrochloride administration (Lin et al., 2007).

Enhanced contraction of arteries in type 2 diabetic mice is chiefly mediated by smooth muscle signaling pathway alterations that modulate calcium sensitivity of contractile proteins, rather than an alteration in endothelial-derived vasoactive factors or special 5-HT receptor subtypes (Nelson et al., 2012). 5-HT causes contraction *via* the activation of both 5-HT_2A_ and 5-HT_2B_ receptors in both diabetic and control mice. 5-HT_1B_ receptor expression is detectable, but does not participate in the contraction in response to 5-HT in either diabetic or control mice, because there is no response to the 5-HT_1B_ receptor agonist CP93129 (Nelson et al., 2012). Aberrant endothelial factors modulated by nitric oxide synthase and cyclooxygenase are also not involved in the increased contractions in response to 5-HT since endothelium removal; similarly, nitro-L-arginine and indomethacin do not have any obvious impact on contractions in response to 5-HT in the aorta in either diabetic or control mice. Contractions increase in response to all 5-HT_2A_ and 5-HT_2B_ receptor agonists, and suppression of both 5-HT_2A_ and 5-HT_2B_ receptors inhibits contraction response to 5-HT in arteries from diabetic mice. This occurs due to altered expression of either receptor subtype in diabetic arteries (Nelson et al., 2012). Contractions in response to 5-HT and both 5-HT_2A_ and 5-HT_2B_ receptor agonists are diminished by inhibiting Rho kinase. Receptor-independent contractions of diabetic arteries to KCl are also enhanced and partly mediated by Rho kinase (Nelson et al., 2012). In most arteries, serotonin-induced contractions are mediated by 5HT_2A_ receptors on vascular smooth muscle, which activates the Rho/Rho kinase pathway to regulate phosphorylation of myosin light chain phosphatase and myosin light chain (Dreja et al., 2002; Nuno et al., 2007). These results suggest that an alteration in rho kinase modulation of calcium sensitivity, but not 5-HT receptor expression, may mediate the hypercontractility of arteries from diabetics.

### 5-HT_1B_ receptors in peripheral arterioles

Cardiopulmonary bypass is linked to a decline in the contractile response of peripheral arterioles to 5-HT, which is exacerbated in diabetes. This 5-HT-stimulated contractile response of peripheral arterioles is mediated by the 5-HT_1B_ receptor in both non-diabetic and diabetic patients (Sabe et al., 2018).

### 5-HT_2A_ receptor antagonists in thrombosis

Polyethylene tube-induced thrombus formation was significantly elevated in streptozotocin-induced diabetic rats compared to that in normal rats (Yamada et al., 2012). Both 5-HT and high glucose upregulate the expression of vascular cell adhesion molecule-1 in human umbilical vein endothelial cells, and this upregulation is further enhanced by the combination of 5-HT and high glucose. Sarpogrelate, a 5-HT_2A_ receptor antagonist, but not aspirin, inhibits the increased expression of vascular cell adhesion molecule-1 triggered by 5-HT and high glucose (Yamada et al., 2012). These results imply that 5-HT drives the increased thrombogenesis in diabetes and that 5-HT_2A_ receptor antagonists may be potential novel candidates to treat diabetic complications.

### 5-HT receptors in retinal superoxide generation

Reactive oxygen species play a crucial role in the pathogenesis of diabetic retinopathy. Incubating cells or retinal explants in 30 mmol/L glucose significantly activated superoxide generation compared to incubation in 5 mmol/L glucose (Du et al., 2015). This response is diminished or blocked by pharmacological inhibition of the α1-adrenergic receptor (a G_*q*_-coupled receptor) or G_*s*_-coupled 5-HT receptors (5-HT_4_, 5-HT_6_, and 5-HT_7_) or by activation of the G_*i*_-coupled α2-adrenergic receptor. Links between different G protein-coupled receptor pathways regarding superoxide generation may originate from hyperglycemia-induced elevations in cytosolic Ca^2+^ levels (Du et al., 2015).

### 5-HT in diabetic vasculopathy

Levels of blood 5-HT in diabetic retinopathy patients are significantly lower than those in non-diabetic retinopathy patients (Pietraszek et al., 1992), while plasma 5-HT concentration is significantly elevated in diabetes. This is linked to vascular alterations in the retina. Platelets in diabetic patients take up less 5-HT than those in non-diabetic patients, spontaneous secretion of 5-HT from platelets is concomitantly enhanced, and platelets show an increased response to 5-HT. However, 5-HT-stimulated aggregation is not related to the presence of retinopathy (Pietraszek et al., 1992). These data indicate that 5-HT may be involved in the pathogenesis of diabetic vasculopathy.

### Role of selective serotonin reuptake inhibitors in diabetes-related cardiovascular complications

Escitalopram, a selective serotonin reuptake inhibitor, improves high-fat/high-fructose diet- and streptozotocin-induced metabolic and cardiac impairment characteristics, such as inflammation, oxidative stress, apoptosis, fibrosis, hypertrophy, and impaired conduction (Ahmed et al., 2020). These benefits may be secondary to its primary beneficial effects on blood glucose control and, consequently, the downregulated expression of receptor for advanced glycation end-product. Escitalopram can be deemed a profitable antidepressant drug in diabetic patients because it ameliorates blood glucose control in diabetes and can also prevent diabetes-related cardiovascular complications (Ahmed et al., 2020).

## Roles of the serotonergic syste in diabetic peripheral neuropathy

**FIGURE 2:**
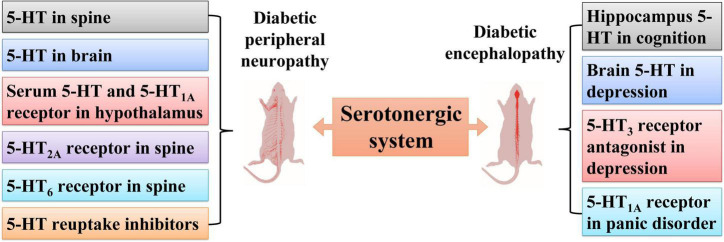
Roles of the serotonergic system in diabetic peripheral neuropathy and diabetic encephalopathy. Serotonergic systems involving 5-HT in serum, spine, brain, the 5-HT_2A_ receptor and 5-HT_6_ receptor in the spine, and the 5-HT_1A_ receptor in the hypothalamus participate in diabetic peripheral neuropathy. The serotonergic system is also involved in diabetic encephalopathy, including hippocampal 5-HT in cognition dysfunction, brain 5-HT in depression, 5-HT_3_ receptor antagonists in depression, and the 5-HT_1A_ receptor in panic disorder.

Hyperglycemia is no longer regarded as a unique etiological factor for diabetic peripheral neuropathy (Nelson et al., 2012; Sabe et al., 2018), as painful neuropathy is also a common comorbidity in patients with diabetes. The roles of the serotonergic system in diabetic peripheral neuropathy and diabetic encephalopathy are described in Figure 2.

### Spinal 5-HT

Painful diabetic neuropathy activates neuronal hyperactivity in the periaqueductal gray and spinal cord. As a critical region in descending nociceptive regulation, the periaqueductal gray makes use of relay stations in the noradrenergic and serotonergic brainstem regions. Streptozotocin-induced diabetic rats present with chemical allodynia and mechanical hyperalgesia, coupled with higher spinal levels of noradrenaline and 5-HT and more neurons expressing tryptophan hydroxylase in the rostral ventrolateral medulla and tyrosine hydroxylase in the A_5_ noradrenergic cell group (Morgado et al., 2011). Administration of insulin growth factor 1 prevents the behavioral signs of painful diabetic neuropathy and restores the neuronal hyperactivity in the spinal cord and ventrolateral periaqueductal gray and the neurochemical alterations in the brainstem and spinal cord. Based on the facilitatory role of noradrenergic and serotonergic descending regulation during chronic pain, the elevated 5-HT and noradrenaline innervation of the dorsal horn in streptozotocin-induced diabetic rats may explain the pain reinforcement during painful diabetic neuropathy. The protective effects of insulin growth factor 1 in painful diabetic neuropathy are likely due to the inhibition of increased peripheral input to the somatosensory system, but direct central effects cannot be excluded (Morgado et al., 2011).

### Serum 5-HT and 5-HT_1A_ receptors in the hypothalamus

Zucker diabetic fatty (fa/fa) rats progress to type 2 diabetes spontaneously with aging and exhibit nociceptive hypersensitivity at 13-week of age. Daily 30-min transcutaneous auricular vagus nerve stimulation (2/15 Hz, 2 mA) for 27 consecutive days significantly inhibited the progression of nociceptive hypersensitivity in Zucker diabetic fatty rats, as measured based on mechanical allodynia and thermal hyperalgesia in the hindpaw. This advantageous impact on nociceptive behavior is associated with an increase in 5-HT plasma levels and upregulated expression of the 5-HT_1A_ receptor in the hypothalamus (Li et al., 2018).

### 5-HT_2A_ receptors in the spine

Systemic or intrathecal injection of duloxetine, a dual inhibitor of serotonin and norepinephrine reuptake, alleviates tactile allodynia in diabetic rats (Mixcoatl-Zecuatl and Jolivalt, 2011). The effect of systemic duloxetine administration is inhibited by intrathecal injection of ketanserin (a non-selective 5-HT_2A_ receptor antagonist) or pruvanserin (a selective 5-HT_2A_ receptor antagonist), suggesting the involvement of spinal 5-HT_2A_ receptors in the mechanism of action of duloxetine. Compared with spinal injection, local peripheral and systemic administration of ketanserin or pruvanserin relieve tactile allodynia in diabetic rats. This effect is restored immediately after local or systemic administration of 1-(2,5-dimethoxy-4-iodophenyl)-2-aminopropane hydrochloride, a 5-HT receptor agonist (Mixcoatl-Zecuatl and Jolivalt, 2011). These data suggest a role of spinal 5-HT_2A_ receptors in the effect of duloxetine in improving painful diabetic neuropathy. These results also show that the role of 5-HT_2A_ receptors relies on the neuraxis level where activation occurs, with peripheral activation resulting in tactile allodynia in diabetic rats; conversely, spinal activation of this receptor relieves tactile allodynia.

Lower urinary tract impairment is one the most common diabetic complications. The 5-HT_2A_ receptor in spinal micturition control is involved in urethane anesthetized diabetes rats. Intravenous injection of the 5-HT_2A_/_2C_ receptor agonist (2,5-methoxy-4-iodoamphetamine) triggers high frequency oscillations and ameliorates micturition (Tu et al., 2015). Expressions of 5-HT_2A_ receptor and 5-HT_2C_ receptor are raised in lumbosacral cord motoneurons, and the number of serotonergic paraneurons are declined in the urethra in diabetes rats (Cao et al., 2019). The function of lower number of urethral paraneurons in diabetes still needs to be clarified but may be associated with the decreased urethral sensation induced by diabetes.

### 5-HT_6_ receptors in the spine

The 5-HT_6_ receptor accurately regulates critical neuro-developmental processes, such as migration and differentiation of neuron (Chaumont-Dubel et al., 2020). The activation of 5-HT_6_ receptors leads to the activation of adenylyl cyclase and may have an excitatory efficiency on neuronal activity (Ruat et al., 1993). Although there are very few studies on the role of 5-HT_6_ receptors in nociception, these receptors seem to mediate pronociceptive efficacies in the spinal cord and the periphery. Pharmacological suppression of 5-HT_6_ receptors diminishes formalin-stimulated nociceptive behavior (Finn et al., 2007), and spinal and local peripheral 5-HT_6_ receptors have a pronociceptive role in formalin-triggered pain (Castaneda-Corral et al., 2009).

Spinal nerve damage results in a loss of descending serotonergic neurons (Leong et al., 2011); in contrast, it does not alter spinal 5-HT_6_ receptor expression (Morgado et al., 2011). Differentiation from nerve injury-caused neuropathy, and 5-HT_6_ receptor expression is obviously downregulated in diabetes-induced neuropathy, which can be due to the effect of intrathecal injection of 5-HT_6_ receptor antagonist SB-258585 to ameliorate thermal hyperalgesia in diabetic rats (Sari et al., 2019). Systemic, but not spinal, inhibition of 5-HT_6_ receptors relieved thermal hyperalgesia in diabetic mice. These results indicate that 5-HT_6_ receptor antagonists may be therapeutic targets for pharmacotherapy of diabetic neuropathy (Sari et al., 2019).

### Selective 5-HT reuptake inhibitors

The antiallodynic efficacies of intrathecal injections of the antidepressants milnacipran, paroxetine, and fluvoxamine were measured in two rat models of neuropathic pain—streptozotocin-induced diabetic neuropathy and chronic sciatic nerve constriction injury (Ikeda et al., 2009). Intrathecal administration of milnacipran, a serotonin and noradrenaline reuptake inhibitor, has antiallodynic efficacy in both chronic constriction injury and streptozotocin-induced diabetic rat models in a dose-dependent manner. Intrathecal injection of both fluvoxamine and paroxetine, which are selective 5-HT reuptake inhibitors, has antiallodynic effects in streptozotocin-induced diabetic rats in a dose-dependent manner, but elicited little antiallodynic effects in rats with chronic constriction injury (Ikeda et al., 2009). These results suggest that the two antidepressants (paroxetine and fluvoxamine) may be effective for the treatment of diabetic neuropathic pain.

## Role of the serotonergic system in diabetic encephalopathy

### Hippocampal 5-HT in cognitive impairment

Rats with streptozotocin-induced type 1 diabetes exhibit cognitive dysfunction during acquisition sessions and long-term retention in the active avoidance test. Diabetic rats also exhibit cognitive dysfunctions in terms of spatial learning, reference, and working memory in the Morris water maze. Streptozotocin remarkably reduced norepinephrine levels in the cortex and dopamine levels in the hippocampus, but increased the levels of 5-HT and dopamine in the cortex 35 days after injection. The 5-HT level in the hippocampus was also significantly elevated (Lin et al., 2018). As a microbial deamination metabolite of tryptophan, 3-indolepropionic acid is an effective neuroprotective antioxidant. Administration of both 3-indolepropionic acid and 5-HT significantly improve cognitive dysfunction in diabetic mice (Liu et al., 2020). Moreover, higher plasma levels of 3-indolepropionic acid are associated with a lower risk of type 2 diabetes (Tuomainen et al., 2018).

### Antidepressant-like action of brain 5-HT

Neuropathological depletion of brain monoaminergic activity, due to chronic diabetes, specifically with regard to the 5-HT system, may result in mood- and behavior-related complications that further reduce the quality of life. Hypothalamic-pituitary-adrenal activity, insulin signaling, and glycogen synthase kinase 3 modulations are hampered and interlinked to the serotonergic system following diabetic progression (Prabhakar et al., 2015).

Disorder of the serotonergic system is a sign of diabetes-related depression. Reduced central 5-HT levels can be mediated by diabetes-induced inflammation and oxidative stress in the central nervous system. Oxidative stress results in dysfunction of the glucocorticoid receptor, negative feedback impairment, and altered functioning of the HPA axis. Hyperactivity of the HPA axis increases glucocorticoid secretion, which in turn inhibits 5-HT synthesis (Prabhakar et al., 2015). On the contrary, neuroinflammation in diabetes modulates the secretion of cytokines, which decreases 5-HT synthesis, increases 5-HT reuptake, and finally reduces its availability (Felger and Lotrich, 2013). In diabetic rats, the levels of 5-HT and brain-derived neurotrophic factor in the cortex and hippocampus are reduced, and serum corticosterone levels are elevated, which suggests hyperactivity of the HPA axis.

A single facial injection of botulinum neurotoxin A induces a quick and extended amelioration of depression-like behaviors in naïve and space-restriction-stressed mice, with reduced duration of immobility in behavioral despair tests. Botulinum neurotoxin A significantly increases the 5-HT levels in numerous brain areas, such as the hippocampus and hypothalamus, in space-restriction-stressed mice (Li et al., 2019). It was also shown to increase the expression of *N*-methyl-D-aspartate receptor subunits NR1 and NR2B in the hippocampus, which are significantly downregulated in space-restriction-stressed mice. Furthermore, botulinum neurotoxin A significantly increases the expression of brain-derived neurotrophic factor in the hypothalamus, hippocampus, amygdala, and prefrontal cortex, which are downregulated in space-restriction-stressed mice. Botulinum neurotoxin A also momentarily upregulates the expression of phosphorylated extracellular signal-regulated kinase and phosphorylated cAMP-response element binding protein, which are downregulated in the hippocampus of space-restriction-stressed mice (Li et al., 2019). These data demonstrate that botulinum neurotoxin A administration has an antidepressant-like effect on mice, which is linked to elevated 5-HT levels and the activation of brain-derived neurotrophic factor/extracellular signal-regulated kinase/cAMP-response element binding protein pathways in the hippocampus, backing further research on botulinum neurotoxin A treatment in depression.

### 5-HT_3_ receptor antagonists in diabetes-induced depression

5-HT is one of the most frequently imbalanced neurotransmitters in the etiology of major depressive disorder, and this system is the chief target of most medications for the treatment of depression (Yohn et al., 2017). Antidepressants treatment in adults increases the risk of developing new-onset type 2 diabetes. Proof from human and animal studies indicates a relationship between increased risk of diabetes and the use of antidepressants that act on 5-HT signaling, such as 5-HT-norepinephrine reuptake inhibitors, 5-HT antagonist and reuptake inhibitors, and noradrenergic and specific serotonergic antidepressants (De Long et al., 2015). Spontaneously Diabetic Torii fatty rats exhibit depression-like behaviors and altered baseline HPA activity. In addition, neurotransmitter levels are dysregulated in the brain areas involved in the pathophysiology of depression (Sakimura et al., 2018).

Diabetic mice also show remarkable behavioral deficiencies, such as anxiety-like behavior in the open field test, depression-like behavior in the forced swim test, and sociability deficits in the social interaction test, accompanied by an obvious decline in 5-HT content in the associated brain areas (Gupta et al., 2016). Similar to fluoxetine, a selective serotonin reuptake inhibitor, *N*-(3-chloro-2-methylphenyl)quinoxalin-2-carboxamide (1 mg/kg), prevents these behavioral deficits and normalizes brain 5-HT levels. *N*-(3-chloro-2-methylphenyl)quinoxalin-2-carboxamide (0.5 mg/kg) ameliorated only diabetes-induced depressive-like behavior and 5-HT decrease, but not anxiety-like effects. 1-(m-chlorophenyl)-biguanide, a 5-HT_3_ receptor agonist, blunted the *N*-(3-chloro-2-methylphenyl)quinoxalin-2-carboxamide-activated behavioral response and elevated brain 5-HT levels (Gupta et al., 2016). These results suggest that *N*-(3-chloro-2-methylphenyl)quinoxalin-2-carboxamide prevents diabetes-induced depressive phenotypes in mice, which may be attributed to the antagonism of 5-HT_3_ receptors and elevation in 5-HT levels in different brain areas.

### 5-HT_1A_ receptors in panic disorder in type 1 diabetes

Diabetic animals exhibit dysregulation of the serotonergic system in some brain regions linked to anxiety-like responses. In one study, 4 weeks after diabetic identification, the threshold of electric stimulation of the dorsal periaqueductal gray to reduce escape behavior in diabetic rats was found to be lower than that in normoglycemic rats (Gambeta et al., 2017); open-field test results indicated that there were no negative effects on locomotor activity in diabetic rats compared with normoglycemic rats. Intra-dorsal administration of the 5-HT_1A_ receptor agonist (±)-8-hydroxy-2-(di-n-propylamino)tetralin in the periaqueductal gray increases the increment threshold in both diabetic and normoglycemic rats, indicating a panicolytic-like effect. Diabetic rats present with a more significant panicolytic-like response than normoglycemic rats, since a higher increment threshold was measured after 8-hydroxy-2-(di-n-propylamino)tetralin treatment, which could be a result of the upregulated expression of 5-HT_1A_ receptors in the dorsal periaqueductal gray in diabetic rats (Gambeta et al., 2017). These data suggest that a lack of serotonergic regulation of the dorsal periaqueductal gray is involved in initiating the panic attacks and that 5-HT_1A_ receptors might be critical for the panicolytic-like response.

## Conclusion

There are two serotonergic systems in the human body—the central system and the peripheral system. With regard to the central serotonergic system, elevating 5-HT signaling has been used to therapeutically reduce body weight by inhibiting appetite. 5-HT is also thought to function in vasospasm and increase platelet aggregability, which can cause atherosclerosis. In peripheral tissues, inhibiting 5-HT signaling may represent a novel anti-obesity treatment by reducing energy expenditure and ameliorating insulin resistance (Crane et al., 2015; Oh et al., 2015). Systemic tryptophan hydroxylase 1 inhibitors and a peripheral tryptophan hydroxylase 1 inhibitor (LP-533401) have been patented for use in treating diabetes and obesity (Kolodziejczak et al., 2015; Abg Abd Wahab et al., 2019). However, there are still no clinical trials on the use of these drugs for the treatment of diabetes.

Upregulated expression of 5-HT_2C_ receptors in both β-cells and the hypothalamus can be a protective strategy to avoid redundant energy intake. 5-HT_2C_ receptor-expressing pro-opiomelanocortin neurons are required to regulate energy and glucose homeostasis (Berglund et al., 2013). Central 5-HT_2C_ receptors control glucose homeostasis and could be a rational target for the treatment of type 2 diabetes. 5-HT_2A_ receptors are broadly expressed in the central nervous system (Zhang and Stackman<suffix>Jr.</suffix>, 2015) and are needed for peripherally administered liraglutide to decrease feeding and body weight (Anderberg et al., 2017). Brain serotonergic deficiencies are linked to depressive-like behavior in diabetes and selective serotonin reuptake inhibitors prevent these behavioral deficits and normalizes brain 5-HT levels (Gupta et al., 2016).

There is limited options for the investigation of the role of central serotonergic system in diabetes in humans *in vivo*. The data in the present review are focused mostly on the results obtained from different animal models of diabetes. More experimental data on humans are expected to support this view in the future.

## Author contributions

JZ, YC, and XL conceptualized the topics. JZ, YC, XL, and HZ wrote and revised the manuscript. All authors contributed to the article and approved the submitted version.

## Conflict of interest

The authors declare that the research was conducted in the absence of any commercial or financial relationships that could be construed as a potential conflict of interest.

## Publisher’s note

All claims expressed in this article are solely those of the authors and do not necessarily represent those of their affiliated organizations, or those of the publisher, the editors and the reviewers. Any product that may be evaluated in this article, or claim that may be made by its manufacturer, is not guaranteed or endorsed by the publisher.
